# Desmoplastic Trichoepithelioma: Histopathologic and Immunohistochemical Criteria for Differentiation of a Rare Benign Hair Follicle Tumor From Other Cutaneous Adnexal Tumors

**DOI:** 10.7759/cureus.9703

**Published:** 2020-08-12

**Authors:** Jawaria Rahman, Muhammad Tahir, Hassan Arekemase, Salikh Murtazaliev, Snehal Sonawane

**Affiliations:** 1 Pathology, Case Western Reserve University School of Medicine, Cleveland, USA; 2 Anatomical and Clinical Pathology, Saint Barnabas Medical Center, Livingston, USA; 3 Internal Medicine, California Institute of Behavioral Neurosciences & Psychology, Fairfield, USA; 4 Pathology, South Bend Medical Foundation, South Bend, USA

**Keywords:** desmoplastic trichoepithelioma, microcystic adnexal carcinoma, syringoma, morphea basal cell carcinoma, cutaneous metastatic breast carcinoma

## Abstract

Desmoplastic trichoepitheliomas (DTEs) are benign cutaneous neoplasms that originate from the hair follicle and exhibit a preference for the facial region. This type of neoplasm is characterized by accelerated growth, with vigorous histologic and immunohistochemical features that may be confused with other skin cancers. Thus, the objective of this study is to establish a definitive diagnosis that can be widely used. This review was systematically carried out and includes case series and studies to establish valuable data that can be used for research. The articles were sought in PubMed, MEDLINE, and Google Scholar using the keywords “desmoplastic trichoepithelioma,” “morphea basal cell carcinoma,” “microcystic adnexal carcinoma,” “syringoma,” and “cutaneous breast carcinoma.” From a total of 65 journal articles, we chose 42 studies describing the clinical features, etiology, histopathology, and immunohistochemical characteristics of tumors. After quality assessment, 10 studies were selected, representing the differentiating features among the four mentioned cutaneous tumors. The differential diagnosis of DTE also includes other cutaneous and follicular tumors. At present, there is no standardized grading system for trichogenic tumors, although several symptomatic terms have been offered. More recently, immunohistochemistry and histopathological studies support the differentiation between the above-mentioned cutaneous tumors. However, additional research needs to be conducted to obtain complete information regarding the specific distinct traits of the indicated cutaneous tumors.

## Introduction and background

Desmoplastic trichoepithelioma (DTE) is an uncommon benign appendageal skin cancer with an incidence of two per 10,000 and amounts to less than 1% of all cutaneous tumors [1,2]. It is a clear-cut version of trichoepithelioma because of its unique clinical and histopathological features [3]. It usually presents as a single lesion, although in some exceptional cases, patients with numerous lesions have also been reported [4,5]. The majority of DTE appears as white to yellowish, hard or soft annular nodule or papule with a central indentation. Female patients are mainly affected, and the usual location is on the face or cheeks. DTE is a unique tumor because of its non-neoplastic nature, histopathological presentation, lack of ulceration, and superficial invasion, along with its development in the young. Like other skin cancers, DTE develops very slowly during the early growth phase and then becomes a stable lesion [6].

DTE exhibits bimodal age distribution and commonly occurs in young children or adults. Research has shown that there is some genetic predisposition for developing trichoepithelioma; routine monitoring would perhaps help with earlier diagnosis. There are usually no symptoms that accompany the lesion, but over time, it may gradually increase in size and shape. Although it is uncommon, trichoepithelioma can exhibit malignant transformation to trichoblastic carcinoma or basal cell carcinoma (BCC) [7].

The diagnosis of DTE may sometimes be problematic, even when evaluated by an expert, and especially when the tumor emulates other benign and malignant tumors. DTE may clinically and histopathologically mimic morphea BCC (MBCC), syringoma, conventional trichoepithelioma, microcystic adnexal carcinoma (MAC), and other tumors. In contrast, histological findings, together with clinical features, may be valuable in making a conclusive diagnosis of some of these lesions [8,9]. DTE can be found during routine skin cancer evaluations, and most of the time, it may have been present on the skin for many years without exhibiting any change or symptoms. Skin cancer screening as a part of physical examinations, follow up and biopsy and consistent monitoring are essential. Although the clinical diagnosis is often complicated and requires expertise in histopathology, a skin biopsy is indicated depending upon the specific location of the lesion, change in morphology, or growth of the tumor. The pathology report determines if the lesion should be observed or followed over time or surgically removed. Hence, a preliminary skin biopsy is essential and required for the correct diagnosis and to determine the most optimal treatment options for a patient.

Surgical excision is the treatment of choice. At the same time, Mohs microscopic surgery is preferred for lesions, especially on the face [3]. Microscopically and clinically, it may be challenging to distinguish DTE from other cutaneous adnexal neoplasms. However, past studies have shown the various differentiations between DTE and other cutaneous cancers. Additional analyses are required to formulate the definitive diagnostic clinical features of cutaneous adnexal tumors. The objective of this study is to summarize as much information as possible from already existing data so the diagnostic features of DTE can be accurately distinguished from other similar skin lesions.

## Review

Methods

We systematically conducted this study using the Preferred Reporting Items for Systematic Reviews and Meta-Analyses [10]. We used different resources to obtain data, yet mainly chose PubMed for collecting most of the information. Resources such as MEDLINE, PubMed Central, WebMD, and Google Scholar were also searched. The keywords used for searching included “desmoplastic trichoepithelioma,” “morphea basal cell carcinoma,” “microcystic adnexal carcinoma,” “syringoma,” and “cutaneous metastatic breast carcinoma.” We focused on the adult population from all over the world without discrimination between gender, race, nationality, and ethnicity. However, several articles that indicated a correlation among various age groups were also added. Among all relevant articles, a quality assessment check was performed using the A MeaSurement Tool to Assess Systematic Reviews checklist, and multiple articles were omitted [11]. We utilized the full-text version of the articles, and the review was scientifically and ethically performed.

Results

We retrieved 134 PubMed and 1,970 Google Scholar studies applying the keywords “desmoplastic trichoepithelioma.” A combination of keywords “desmoplastic trichoepithelioma” and “morphea basal cell carcinoma” provided 80 PubMed articles; “desmoplastic trichoepithelioma” and “microcystic adnexal carcinoma” yielded 33 PubMed articles; “desmoplastic trichoepithelioma” and “syringoma” provided 134 articles; and the last combination of keywords, “desmoplastic trichoepithelioma” and “cutaneous breast carcinoma,” resulted in only five suitable articles. A total of 65 articles were selected from these findings. Among the selected studies, most of the investigations were not evidence-based. After executing the exclusion/inclusion criteria and removing the duplicates, we obtained 42 articles that were considered for review. Most of the related articles that were chosen were case studies and series that described the clinical presentation and histopathological findings of the indicated adnexal tumors. After much consideration, 10 contextual studies were chosen because they showed the distinct differentiation features between the indicated cutaneous adnexal tumors. Table 1 lists the included studies [12-22].

**Table 1:** Relevant studies showing a distinct differentiation between the DTE and indicated cutaneous adnexal tumors. AR, androgen receptor; CEA, carcinoembryonic antigen; basal cell carcinoma, BCC; CK, cytokeratin; DTE, desmoplastic trichoepithelioma; EGFR, epidermal growth factor receptor; EMA, epithelial membrane antigen; FAP, fibroblast activation protein; MAC, microcystic adnexal carcinoma; MBCC, morphea basal cell carcinoma; PHLDA1, pleckstrin homology-like domain family A member 1; PKK1, pan-cytokeratin.

Study	Purpose of the Study	Tumor of Differentiation	Result	Conclusion
Katona TM et al., 2008 [12]	To study a CK20 and AR antibodies panel to distinguish DTE from morphea form/infiltrative BCC.	MBCC	AR expression was observed in DTE 13% (2/15) and MBCC 65% (20/31). CK20-positive Mërkel cells were found in 100% (15/15) of DTE and 3% (1/31) of MBCC. In 87% (13/15) of DTE cases, the predicted pattern of AR-, CK20+ immunophenotype was present. In MBCC cases, AR+, CK20- was 61% (19/31). No DTE was AR+, CK20- and no AR-, CK20 + was an MBCC.	Immunohistochemical AR and CK20 stains are useful for distinguishing DTE from MBCC. The immunophenotype AR-, CK20 + is sensitive (87%) and DTE-specific (100%). For MBCC, the AR+, CK20- immunophenotype is specific (100%) and moderately sensitive (61%).
Sellheyer K et al., 2011 [13]	To study MBCC and DTE for PHLDA1, a follicular stem cell marker.	MBCC	Excluding one case, all 16 desmoplastic trichoepitheliomas were immunoreactive to PHLDA1 with more than 80% of the cells stained, while all 14 MBCCs were PHLDA1 negative except for ulcerated tumors. In the latter, the near-ulcer tumor islands were PHLDA1 positive, while the deeper portions of the tumor remained immunonegative.	The hair follicle bulge marker PHLDA1 distinguishes between DTE and nonulcerated MBCC.
Abbas O et al., 2010 [14]	To investigate fibroblast-activation protein: a single marker that confidently distinguishes morphea/infiltrative BCC from DTE.	MBCC	Microscopically, differentiation of DTE from morphea form/infiltrative BCC can be difficult because both show the islands and strands of basaloid cells embedded in a sclerotic stroma. A type II membrane-bound glycoprotein, FAP, which is part of the serine protease family, has been shown to heal wound granulation tissue. Expression of FAP was noted in peritumoral fibroblasts for all instances of morphea form/infiltrative BCC (25 of 25, 100%) but not in DTE (0 of 25, 0%).	Application of the fibroblast-activating protein as a histological component confidently distinguishes morphea form/infiltrative BCC from DTE.
Tse JY et al., 2013 [15]	A comparative study of MAC versus DTE.	MAC	Investigated the histological characteristics of 30 MAC and 39 DTE cases and conducted immunostains of 20 MACs and 18 DTEs with CK17, CK19, and EGFR. MAC cases occurred in older patients versus DTE (median, 67 years vs. 34 years). CK19 appears to be a useful adjunct because its expression was seen in 70% (14/20) of MAC vs. 22% (4/18) of DTE cases. However, due to the overlapping immune profile, clinical use may be limited in individual cases. In all MAC and DTE cases, the expression of CK17 and EGFR was observed.	CK19 helps distinguish between MAC and DTE.
Sellheyer K et al., 2013 [16]	To study the differentiating points of MAC, DTE, and MBCC using PHLDA1 and stem cell markers.	MAC	Sixteen of 21 DTE samples were immunoreactive to histologic stain for antibodies to the epithelial cell adhesion molecule BerEP4/EpCAM. All 21 DTEs were PHLDA1 positive. MAC showed a mixed pattern of staining. The expression of CK15 was noted in 20/21 DTE, whereas most MAC cases were CK15 negative. CK19 stained more MAC than DTE.	BerEP4/EpCAM does not distinguish between MAC and DTE. CK15 and CK19 are useful adjuncts for differential diagnosis of adnexal sclerosing neoplasms.
Aslam A, 2017 [17]	MAC, and a list of other unusual adnexal malignancies	MAC	The study showed that MAC is negative for BerEP4/EpCAM and positive for CK15. In contrast, BerEP4/EpCAM and CK15 are positive in DTE.	BerEp4/EpCAM can be used to distinguish between MAC and DTE.
QY Wang et al., 2015 [18]	Clinicopathological study of three cases of DTE	Syringoma	This review article referred to the differentiation of histopathological and immunohistochemical markers between DTE and syringoma. Histopathologically, syringoma is identified with uncommon narrow strands of cancer cells with only ductal differentiation and periorbital involvement. However, DTE exhibited continuous narrow strands of tumor cells, keratinous cysts, and epidermal hyperplasia. Also, DTE is mostly solitary with many granuloma and calcification of foreign bodies. Immunohistochemically positive DTE had strong CK20 with negative CEA. The hallmark of syringoma is positive CEA, and rarely positive CK20.	DTE is positive for CK20 and negative for CEA, whereas the opposite is true for syringoma.
Ciarloni L et al., 2016 [19]	A study of the clinicopathological features in 244 cases of syringoma	Syringoma	The study outlined the precise histological presentation of syringomas. Syringomas are mainly located in the reticular dermis with acanthotic skin and basal layer pigmentation. The tumor usually consists of small duct-like structures in the skin.	The study showed remarkable histopathology of syringomas that enabled prominent differentiation between syringomas and DTE.
Mordenti C et al., 2000 [20]	To study the histopathologic and immunohistochemical features of cutaneous metastatic breast cancer	Cutaneous metastatic breast carcinoma	Cutaneous metastatic breast cancer is usually found in the chest, which is atypical for DTE. Histological varieties include glandular, Indian file pattern of neoplastic cells between collagen fibers, malignant cell lymphatic embolization, and fibrotic and epidermotropic patterns. Immunohistochemical studies of skin metastatic breast cancer demonstrated high tumor cell positivity for PKK1 and EMA.	The study demonstrated significant differentiation of metastatic cutaneous breast cancer from DTE based on histopathological features.
Tan AR et al., 2016 [21]	To demonstrate the cutaneous manifestations of breast cancer	Cutaneous metastatic breast cancer	Histopathologically, there are atypical tumor cells and red blood cells present with dilated vascular channels in cutaneous metastatic breast cancer. Furthermore, the skin changes, and warm, tender plaques or patches with well-defined borders appear, which are similar to the skin condition erysipelas. [22]	This investigation described critical differentiation points between metastatic cutaneous breast cancer and DTE based on histopathological features.

Discussion

DTEs, also known as sclerosing epithelial hamartomas, are benign cutaneous neoplasms that originate from the hair follicle [23]. An incidence of DTE of one in 5,000 has been reported in a cohort of British adults [24]. DTEs represent less than 1% of all cutaneous neoplasms and are related to one entity of the diverse spectra of benign follicular differentiated appendageal tumors of the skin [1,2].

Three unique types of DTE have been identified, namely, solitary, multiple, and desmoplastic [10]. DTE is usually noted in middle-aged women but has been more commonly reported in two-mode age groups. It typically does not exhibit any signs or symptoms and often presents as a solitary, indurated, annular, and centrally depressed papule or plaque [6,25]. The most regularly influenced areas are those that are sun-exposed areas, especially facial regions such as the chin, cheeks, and forehead. Less commonly, the tumors may be localized to the upper trunk area, the neck, and the scalp [12]. DTE has a steady growth pattern and slowly grows up to 1 cm in diameter. Multiple lesions are rare [23].

There is some extent of genetic predisposition to developing trichoepithelioma. The chromosomal mutations on 9p21 and 16q12-q13 are considered to be related to DTE. Multiple familial trichoepitheliomas occur because of an autosomal-dominant disorder, marked by positive family history, histopathological characteristics, and numerous papules or nodules [7].

**Figure 1:**
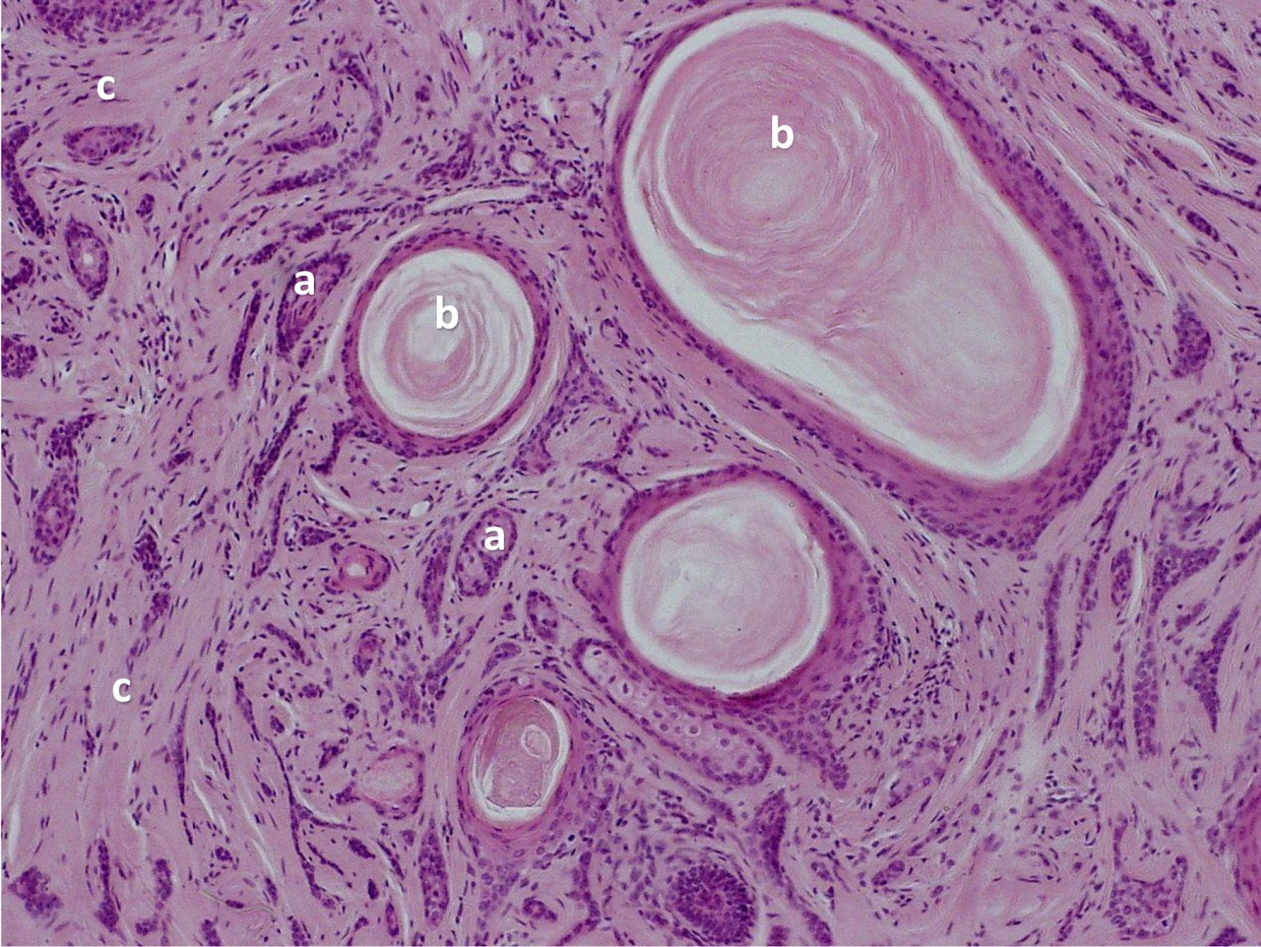
Desmoplastic trichoepithelioma: high-resolution of narrow strands of basaloid cells (a) with keratinized horn cyst (b) and fibrous stroma (c). Used under Creative Commons license CC BY-SA 3.0 by Wozniak and Zielinski [26].

**Figure 2:**
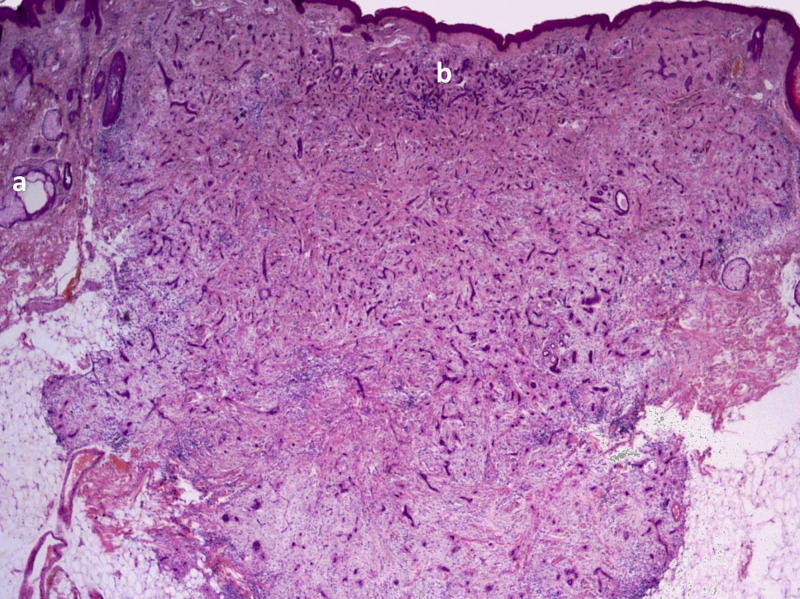
Desmoplastic trichoepithelioma: whole mount view of keratinized horn cyst (a) with basaloid epithelial cords and strands with marginal palisade (b). Used under Creative Commons license CC BY-SA 3.0 by Wozniak and Zielinski [27].

In 1977, Brownstein and Shapiro investigated a series of 49 cases and described the microscopic histologic characteristics of DTE [1]. They observed narrow strands of basaloid tumor cells, keratinous cysts, and a desmoplastic stroma, as shown in Figure 1 and Figure 2 [26,27].

These characteristics have remained a unique triad for the dermatopathology of DTE since Brownstein and Shapiro’s 1977 study [1]. Commonly, DTE lesions are superficial and seldom reach the lower dermis. DTE tends to invade the perineural and intraneural regions, and this has also been found in other cutaneous malignancies [12]. Khelifa et al. also summarized the histopathological findings of DTE, which is usually well-circumscribed, uniform, and confined to the papillary dermis and upper two-thirds of the reticular dermis [28]. Another element of DTE is the presence of horn cysts and frequent calcification. There are no indications of mitotic figures, pleomorphism, or apoptotic activity in the epithelium [18].

Full-thickness skin biopsy is the best choice for diagnosing DTE. Small incomplete biopsies may cause uncertainty because BCC and microcystic adnexal carcinoma may resemble each other. Resampling or re-excision might be essential for the definitive diagnosis or complete eradication in uncertain cases [23]. The treatment of choice for solitary lesions on different parts of the body is surgical excision. For lesions on the face, Mohs microscopic surgery is recommended to obtain clear surgical margins. In the case where multiple lesions require treatment and cancer cells and deep tissue invasion is present, total excision should be performed to exclude malignancy [1].

**Figure 3:**
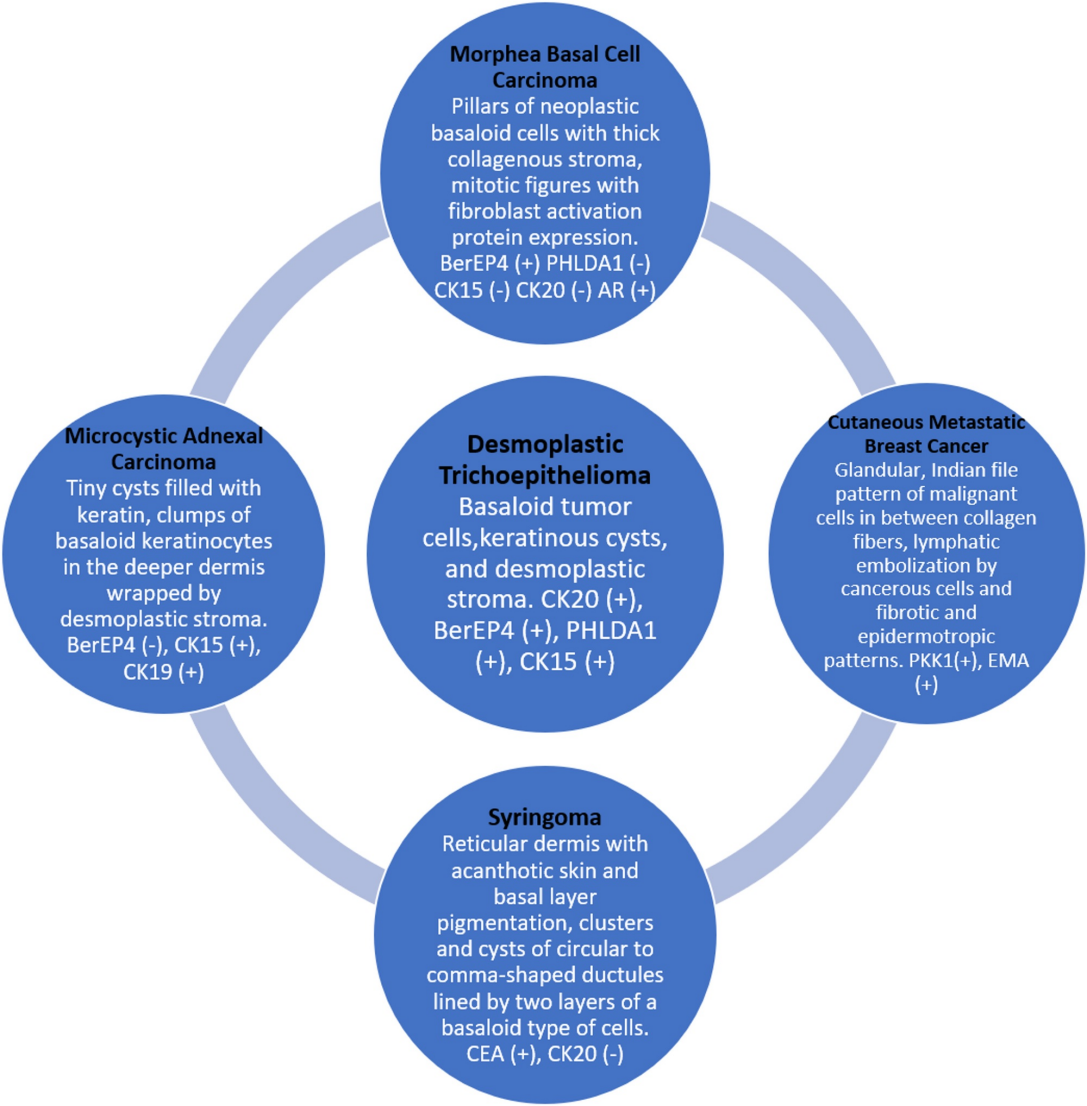
Differentiation between desmoplastic trichoepithelioma and other cutaneous tumors. Abbreviations: AR, androgen receptor; CEA, carcinoembryonic antigen; CK, cytokine; EMA, epithelial membrane antigen; PHLDA1, pleckstrin homology-like domain family A member 1; PKK1, pan-cytokeratin.

Clinically, it is difficult to differentiate DTE from other skin lesions such as MBCC, MAC, syringoma, and cutaneous metastatic breast cancer. However, many studies have been performed to distinguish DTE from other cutaneous adnexal tumors. Additional research is required to understand how DTE can easily be differentiated from other skin cancers according to the histopathological and immunohistochemical aspects. Figure 3 presents the differential points of DTE from the above mentioned cutaneous tumors [12,13,15-18,20].

Morphea Basal Cell Carcinoma

MBCC is the most prevalent skin malignancy in the United States, and the incidence is increasing by 4% to 8% every year because of total sun exposure of individuals and a maturing population [29,30]. However, the occurrence rates of MBCC, metastasis, and age-adjusted death rates are only 0.0028%, 0.5%, and 0.12 per 100,000, respectively [31]. The morphea (sclerosing) form of BCCs has a higher frequency of relapse and perineural invasion. Tumors present as waxy, depressed, scar-like plaques, and are commonly ulcerated [32].

There are clinical and microscopic similarities between DTE and MBCC. Histopathologically, MBCC is especially difficult to differentiate from DTE, particularly in the setting of a small biopsy specimen [33,34]. One study by Katona et al. used the immunohistochemical cytokeratin (CK) 20 and androgen receptor (AR) antibodies to differentiate DTE from MBCC. The study showed that AR expression was seen in 13% of DTE and 65% of MBCC cases. CK20-positive Merkel cells were identified in 100% of DTE and 3% of MBCC cases [12]. Another study by Sellheyer et al. emphasized the role of pleckstrin homology-like domain family A member 1 (PHLDA1, a stem cell marker for differentiation between DTE and MBCC) [13]. The study demonstrated that DTEs were immunoreactive to PHLDA1 in 15 of 16 cases with more than 80% of the cells stained, whereas all 14 MBCCs were PHLDA1‐negative except for ulcerated tumors [13].

Fibroblast activation protein, a type II membrane-bound glycoprotein related to the serine protease family, is expressed in the granulation tissue of healing wounds. Microscopically, differentiating the morphea form/infiltrative BCC from DTE may be difficult because both exhibit strands and islands of basaloid cells embedded in a sclerotic stroma. The expression of fibroblast activation protein was noted in peritumoral fibroblasts in all cases of the morphea form/infiltrative BCC (25 of 25, 100%), but not in any instances of DTE (0 of 25, 0%) [14].

After analyzing the studies mentioned above, we noted that immunohistochemical stains for AR and CK20 are useful to differentiate DTE from MBCC. The AR-, CK20+ immunophenotype is sensitive (87%) and specific for DTE (100%). The AR+, CK20- immunophenotype is specific (100%) and moderately sensitive (61%) for MBCC. Moreover, the hair follicle bulge marker PHLDA1 differentiates between DTE and nonulcerated MBCCs. However, the use of fibroblast activation protein as a histologic adjunct enables accurate differentiation of the morphea form/infiltrative BCC from DTE. Sometimes the diagnosis between DTE and infiltrative BCC is exceedingly difficult, even when assessed by a dermatopathology expert. Therefore, establishing the correct diagnosis is crucial for clinicians, as the first entity represents a benign adnexal tumor with an excellent prognosis, while the latter is a high-risk variant of BCC that requires much more stringent clinical management.

Microcystic Adnexal Carcinoma

MAC is a relatively uncommon cutaneous tumor first depicted by Goldstein et al. in 1982 [35]. It exhibits both follicular and ductal differentiation and is presumed to arise from a pluripotent adnexal keratinocyte with a preference for the head and neck [35-39]. It is a sluggish, deeply infiltrative, and locally invasive tumor with a high affinity for perineural intrusion [40,41]. Histologically, tumors frequently have a benign appearance, especially the superficial parts, which can prompt misdiagnosis as a syringoma or a benign follicular neoplasm [41,42].

A sufficiently deep biopsy of the MAC lesion is required for a proper diagnosis [43]. Tse et al. performed CK17, CK19, and epidermal growth factor receptor (EGFR) immunostains on 20 MACs and 18 DTEs. MAC cases occurred in older patients compared with DTE (median, 67 years vs. 34 years). CK19 seems to be a helpful adjunct because its expression was observed in 70% (14/20) of MAC versus 22% (4/18) of DTE cases. However, the clinical usefulness in individual situations may be limited because of the overlapping immune profile. CK17 and EGFR expression occurred in all analyzed MAC and DTE cases [15]. Sellheyer et al. performed immunohistochemical staining for DTE and MAC. BerEP4/EpCAM was immunoresponsive in 16 of 21 DTE cases, and all 21 DTE cases were PHLDA1 positive. MAC exhibited a mixed staining pattern. CK15 appeared in 20/21 DTE cases, whereas the majority of MAC cases were CK15 negative. CK19-positive staining was observed in more MAC cases than in DTE cases [16]. A study carried out by Aslam et al. revealed that microcystic adnexal carcinoma is BerEP4/EpCAM negative and CK15 positive, while DTE is positive for both BerEP4/EpCAM and CK15 [17].

After analyzing the studies where MAC was accurately differentiated from DTE, it was apparent that CK19 assists with distinguishing between the two conditions. CK15 and CK19 are valuable adjuncts in the differential diagnosis of sclerosing adnexal neoplasms. However, Aslam et al. argued that BerEp4/EpCAM may be a useful differentiator.

Syringoma

Syringomas are generally rare benign eccrine tumors of sweat organs, as witnessed by their incidence rate of nine per 10,000. They are prevalent in women, with the most usual clinical presentation being numerous lesions present on the eyelids. These lesions predominantly develop on the face and trunk and are commonly brown and pruritic, and this similarity in visual appearance to other skin cancers results in difficulty in accurately diagnosing them. Vulvar forms are frequently pruritic, and because they are rare, little is known about them [19].

A retrospective review by Wang et al. emphasized the significant histopathological differentiating points between syringomas and DTE [18]. The study showed that syringomas are found with unusual narrow strands of tumor cells with only ductal differentiation and periorbital involvement. In contrast, DTE exhibits continuous narrow strands of tumor cells, horn cysts, and epidermal hyperplasia. DTE tumors are mostly solitary with numerous foreign body granulomas and calcification. Immunohistochemically, DTE is strongly CK20 positive and carcinoembryonic antigen (CEA) negative. The hallmark of a syringoma is that it is positive for CEA and rarely positive for CK20 [18]. A study conducted by Ciarloni et al. described the detailed histological presentation of syringoma. According to the study, all syringomas are mainly located in the reticular dermis with acanthotic skin and basal layer pigmentation. The tumor explicitly consists of small duct-like structures in the skin [19].

Per our analysis, it is apparent that syringomas possess specific narrow strands of tumor cells with ductal differentiation. In contrast, DTE exhibits continuous narrow strands of tumor cells with horn cysts and epidermal hyperplasia. DTE is mostly solitary, with much foreign body granuloma, whereas syringomas are found in multiple numbers with acanthotic skin.

Cutaneous Metastatic Breast Cancer

Breast cancer is the most frequently diagnosed malignancy in women. After lung cancer, it is the second most common cause of cancer death in women. Approximately 5% to 10% of breast cancer patients present with metastases in their first encounter. Nevertheless, most patients with metastatic disease have a relapse of early-stage breast cancer. Breast cancer may present with cutaneous manifestations, either directly in the form of skin metastases or direct tumor extension, or indirectly, as a paraneoplastic syndrome.

Another critical setting of breast cancer is the presentation of cutaneous metastatic tumors associated with an inherited cancer syndrome called Cowden syndrome. The most common presentation is nodules, found in 80% of patients. The buds are often non-tender, round or oval, mobile, firm, and have a rubbery texture. They can be solitary or multiple, usually flesh colored, but could be brown, bluish-black, or pink to red-brown. They can also become ulcerated and infected. Histologic examination shows solid aggregates of neoplastic cells [21]. Rasch et al. described the skin changes, which were characterized by warm, tender plaques or patches with well-defined borders that are found in cutaneous metastatic breast cancer and are similar to the skin condition erysipelas [22]. Histopathologically, there is an invasion of the dermal lymph vessels by the tumor [22].

Mordenti et al. demonstrated that cutaneous metastatic breast cancer most commonly involves the chest, which is atypical for DTE [20]. Histological variants of cutaneous metastatic breast cancer include glandular, Indian file pattern of malignant cells between collagen fibers, lymphatic embolization by cancerous cells, and fibrotic and epidermotropic patterns. Immunohistochemical investigations showed strong positivity of tumor cells for pan-cytokeratin and epithelial membrane antigen [20]. Tan et al. showed histological points of differentiation of atypical tumor cells and red blood cells present with dilated vascular channels [21].

A summary of the clinical and histological features, immunohistochemistry, and management of DTE, MBCC, MAC, and syringoma is shown in Table 2 [12,13,15-18,20].

**Table 2:** Summary of the clinical and histological features, immunohistochemistry, and the management of DTE, MBCC, MAC, and syringoma. AR, androgen receptor; BCC, basal cell carcinoma; CEA, carcinoembryonic antigen; CK, cytokeratin; DTE, desmoplastic trichoepithelioma; EMA, epithelial membrane antigen; MAC, microcystic adnexal carcinoma; MBCC, morphea basal cell carcinoma; PHLDA1, pleckstrin homology-like domain family A member 1; PKK1, pan-cytokeratin.

Tumor Types	Gross Clinical Features	Histological Features	Immunohistochemistry	Treatment
DTE [12]	Usually symmetrical, hard ring-shaped nodule or plaque with central indentation, horn cyst, calcification, and granuloma formation	Multiplication of basaloid cells organized in tiny clusters and strands in the shallow dermis	CK20 (+), BerEP4 (+), PHLDA1 (+), CK15 (+)	Observation and follow-up
MBCC [13]	Pinkish-white or yellowish scar with fibrosis and central ulceration, presence of significant aggregation and occasional granuloma formation	Slope-shaped pillars of neoplastic basaloid cells with thick collagenous stroma, and mitotic figures with fibroblast activation protein expression	BerEP4 (+), PHLDA1 (-), CK15 (-), CK20 (-), AR (+)	Mohs micrographic surgery
MAC [15]	Asymmetrical, poorly enveloped, solitary infiltrative papule or plaque that has an insidious onset with perineural and perichondral involvement	Tiny cysts filled with keratin, and clumps of basaloid keratinocytes in the deeper dermis wrapped by desmoplastic stroma	BerEP4 (-), CK15 (+), CK19 (+)	Mohs micrographic surgery
Syringoma [18]	Present as numerous 1-5 mm macules and papules that are skin-colored to yellowish-brown on the cheeks and lower eyelids. Rarely found as solitary; distributed mainly on the face, abdomen, chest, and genitals	Stromal fibrosis, very well-circumscribed, with clusters and cysts of circular to comma-shaped ductules lined by two layers of a basaloid type of cells	CEA (+), CK20 (-)	Laser therapy is beneficial, as well as cryotherapy, and excision
Cutaneous metastatic breast cancer [20]	Most commonly presented as skin-colored or pinkish to yellowish big mass or nodule on the chest wall, abdomen or neck	Cellular atypia, glandular, Indian file pattern of malignant cells between collagen fibers, lymphatic embolization by cancerous cells, and fibrotic and epidermotropic patterns	PKK1(+), EMA (+)	Cryotherapy, chemotherapy, and excision

## Conclusions

The results of the current study combined with the already existing data support the viewpoint that DTE is a particularly uncommon benign skin adnexal tumor. Local excision is the treatment of choice. However, an “observe and see” strategy can be used as a management practice in those situations where the clinical properties are unique to DTE. For cancer as rare as DTE, the pieces of evidence for relapse are not reliable; therefore, the specific relapse rate cannot be determined. However, the tumor shares various clinicohistopathological similarities with MBCC, MAC, syringoma, and cutaneous metastatic breast cancer. Immunohistochemical markers and histopathological findings may assist in the differentiation of skin cancers. Regardless, specific diagnostic techniques for the differentiation of skin tumors are still insufficient, and many cases may be left untreated. The diagnosis and differentiation of DTE remain crucial because the treatment and prognosis of other tumors mimicking DTE are different. As a whole, DTE is a rare cutaneous adnexal tumor, and its aggressive histological features can cause diagnostic uncertainty and confusion with other tumors. To distinguish structurally similar but biologically different tumor entities often requires a comprehensive diagnostic approach that includes the complexity of histopathological, immunohistochemical, and clinical findings.
